# Combining Physiological and Neuroimaging Measures to Predict Affect Processing Induced by Affectively Valent Image Stimuli

**DOI:** 10.1038/s41598-020-66109-3

**Published:** 2020-06-09

**Authors:** Kayla A. Wilson, G. Andrew James, Clint D. Kilts, Keith A. Bush

**Affiliations:** Brain Imaging Research Center, University of Arkansas for Medical Sciences, Little Rock, USA

**Keywords:** Neuroscience, Physiology

## Abstract

The importance of affect processing to human behavior has long driven researchers to pursue its measurement. In this study, we compared the relative fidelity of measurements of neural activation and physiology (i.e., heart rate change) in detecting affective valence induction across a broad continuum of conveyed affective valence. We combined intra-subject neural activation based multivariate predictions of affective valence with measures of heart rate (HR) deceleration to predict predefined normative affect rating scores for stimuli drawn from the International Affective Picture System (IAPS) in a population (n = 50) of healthy adults. In sum, we found that patterns of neural activation and HR deceleration significantly, and uniquely, explain the variance in normative valent scores associated with IAPS stimuli; however, we also found that patterns of neural activation explain a significantly greater proportion of that variance. These traits persisted across a range of stimulus sets, differing by the polar-extremity of their positively and negatively valent subsets, which represent the positively and negatively valent polar-extremity of stimulus sets reported in the literature. Overall, these findings support the acquisition of heart rate deceleration concurrently with fMRI to provide convergent validation of induced affect processing in the dimension of affective valence.

## Introduction

The importance of affect processing to human behavior has long driven researchers to pursue its measurement. Affect processing impacts all facets of an individual’s life, from their emotional responses to their perceptual processing^1^ and affect processing responses have been measured using a variety of modalities, from analyses of self-report to neuroimaging. However, in the past researchers have been forced to choose a single modality, thus constraining the interpretation of affective responses^2^. With more recent focus on validity and generalizability, researchers have begun measuring affect processing according to multiple, independent modalities^3^. With hopes to add to this literature, this study examines the simultaneous measurement of perceived affective valence by both neuroimaging and physiology.

## Psychophysiological and behavioral measures of affect processing

Multiple measures exist that detect weak, but reliable, physiological and behavioral responses to affectively coded stimuli. For example, the galvanic skin conductance response (SCR) has been widely employed as a measure of autonomic arousal responses to affectively laden stimuli^4–6^. Meanwhile, activity in the zygomaticus (smile expression) and corrugator (frown expression) muscles, detected via facial electromyography, has been linked, respectively, with observations of positively and negatively valent stimuli^7–12^. Similarly, physiological responses to positively and negatively valent stimuli have been characterized by variations in heart rate deceleration following stimulus onset^7,13–18^. Due to the variation in the dimensions of affect that these psychophysiological measures detect, the use of multiple modalities would contribute to a broader understanding of affective processing.

## Linking Neural Processing Correlates of Affect Processing with Psychophysiological Measures

The advent of functional magnetic resonance imaging (fMRI) of blood oxygen-level dependent (BOLD) signal acquisitions enabled the inference of functional neuroanatomical circuit responses to affective stimuli^19–25^. However, a critical methodological step in these experiments, often omitted, is the verification of affect induction through independent measurement. To this end, patterns of neural activation have been compared to both cardiac responses to categorical emotional faces^26^ and skin conductance responses during reappraisal of negative affectively valent stimuli^27^. Heller *et al*.^3^ demonstrated that concurrent recording of facial EMG could objectively measure the affective valence induced by image stimuli when reporting functional neuroanatomical activations.

More recently, multivoxel pattern analysis (MVPA) has been proposed as a means of characterizing affect induction. MVPA of BOLD signal^28^ was shown to accurately predict the affective content of previously unseen stimuli, based solely on the temporally succinct neural activation patterns that represent specific cognitive processes within the brain^29^. Indeed, growing empirical evidence describes reproducible^30^, distributed^30–33^, and predictive^29,30,33–35^ neural activation patterns that are central to affect processing^31–33^. Indeed, the power of neural activation patterns to predict affect processing in human subjects, particularly in un-tasked experimental domains lacking ground truth^36,37^, calls for rigorous validation of MVPA as a measure of affect induction against long-established measurement standards rooted in physiology and behavior. To address this need, a recent study by the authors^35^ validated affective arousal prediction efficacy of MVPA models versus the galvanic skin conductance response (SCR). Ideally, validation of MVPA predictions would extend to the orthogonal affective dimension of valence. With valence shown to be well-characterized by heart rate deceleration^7,13–18^, and, as heart rate may be recorded via photoplethysmogram (PPG), an optical instrument that minimally interacts with the MRI environment, PPG offers a reliable, widely-used, measure of heart rate that may be acquired concurrently with MRI.

## Heart Rate Change as a Measure of Affective Valence

Heart rate deceleration (also termed HR change and abbreviated throughout this work as ΔHR) is calculated as the difference in heart rate from a baseline value, which can vary by experiment. Recent literature has shown a correlation between HR change, measured relative to the time of affective stimuli, and the valence ratings of those stimuli. Specifically, negatively valent stimuli have been associated with immediate, dramatic deceleration in heart rate^7,13–18^. In contrast, positively valent stimuli have been associated with a triphasic trajectory of HR change, consisting of an immediate deceleration followed by a slight acceleration before decelerating again^14,15,17,38^.

## Aims of Present Study

The aim of this study is to extend the comparison between physiological (i.e., heart rate change) and neural activation measurements of affective valence induction. Specifically, we perform this comparison on a stimulus set representing a broad continuum of conveyed affective valence, i.e., including a range of neutral and weakly valent stimuli in addition to the polar-extremes of positive and negative valent stimuli as have been commonly reported in the literature.

## Materials and Methods

### Study overview

We analyzed data acquired from two studies that share a common image set and ordering of tasks: the Intrinsic Neuromodulation of Core Affect (INCA) study and the Cognitive Control Theoretic Mechanisms of Real-time fMRI-Guided Neuromodulation (CTM) study. Both of these studies are functional neuroimaging explorations of affect perception, unguided affect regulation, and real-time fMRI-guided volitional affect regulation. Details of the relevant tasks have been previously reported in Bush *et al*.^30^. To aid study rigor and reproducibility, we have elaborated below the experimental details for the combined studies related to their task design, image selection algorithm, and data processing methodologies.

All study procedures were conducted in the Brain Imaging Research Center (BIRC) at the University of Arkansas for Medical Sciences (UAMS). All participants provided written informed consent. All study procedures were conducted with approval and oversight by the UAMS Institutional Review Board in accordance with the Declaration of Helsinki and relevant institutional guidelines and regulations.

Participation demands were nearly identical across both studies, occurring in two sessions on separate days, save the following differences. CTM included the Perceived Invalidation of Emotion Scale (PIES) assessment as well as facial EMG measurement concurrent with fMRI. INCA required a negative urine screen prior to day two activities while CTM required a negative urine screen prior to day one and day two activities. During Session 1, participants provided written informed consent, were screened for clinically relevant exclusionary criteria via structured clinical interview (SCID-I/NP), and completed behavioral surveys and questionnaires. Neuroimaging was conducted during Session 2 and lasted approximately 1 hour. This analysis includes only data acquired during the first two functional image acquisitions (i.e., scans) of the session, comprising the System Identification Task (detailed below).

The authors have made the source code used to conduct this analysis publicly available at: https://github.com/kabush/HR.

### Participants

The participant sample (n = 50) used for this analysis completed the System Identification Task within either the INCA (n = 19) or CTM (n = 31) studies. The sample had the following demographic characteristics: age [mean(s.d.)]: 33.7(13.6), range 18‒63; sex: 29 (58%) female; race/ethnicity: 43 (86%) self-reporting as White or Caucasian, 6 (12%) as Black or African-American, 1 (2%) as Hispanic or Latino; education [mean(s.d.)]: 16.5(2.8) years, range 12‒23; WAIS-IV IQ [mean(s.d.)]: 106.6(13.8), range 74‒137. All participants were right-handed, native-born United States citizens (a requisite condition for applying IAPS normative scores), medically healthy, without current Axis I psychopathology including mood disorders (assessed via SCID-IV clinical interview (APA, 1994)), no current reported usage of psychotropic medication, and produced a negative urine screen for drugs of abuse (cocaine, amphetamines, methamphetamines, marijuana, opiates, and benzodiazepines) immediately prior to the MRI scan. Additionally, all participants’ vision was corrected to 20/20 during the MRI scan using a lens system and color-blindness was exclusionary.

### System identification task

**Figure 1:**
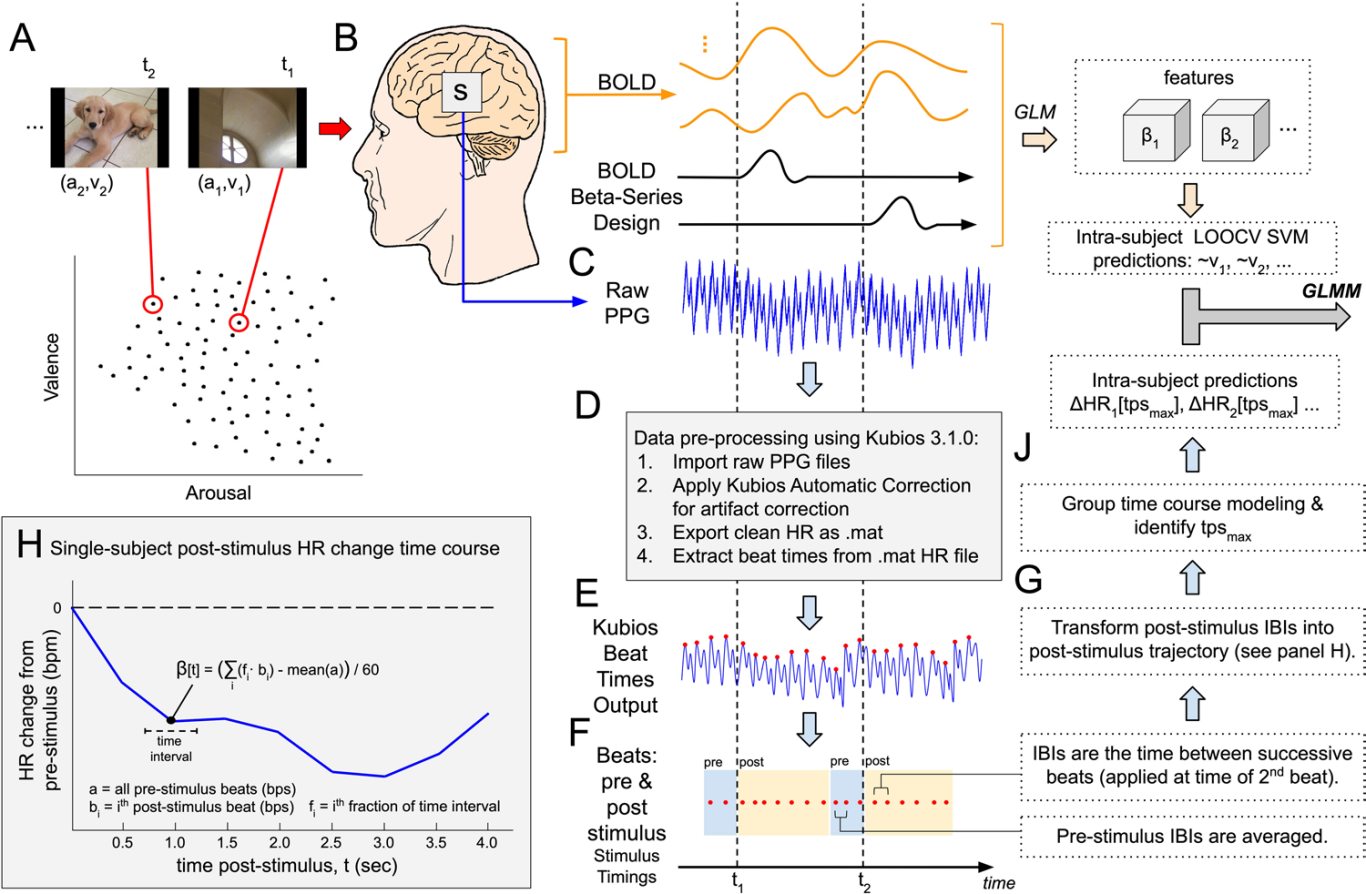
Methodological Overview. Experiment Design: (**A**) Ninety images drawn from the International Affective Picture System (IAPS) were presented for 2 s each interleaved with random inter-trial intervals uniformly randomly sampled on the range of 2–6 s during concurrent recording of both fMRI BOLD and photoplethysmogram (PPG) signals. fMRI processing: (**B**) fMRI BOLD signal was preprocessed to remove noise and motion artifacts, transformed into whole-brain neural activation patterns via General Linear Modeling according to the beta-series method^43^, and then applied to predict normative valence scores according to intra-subject leave-one-out cross validated linear support vector machine learning as reported previously by Bush *et al*.^35^. (**C**) Raw PPG signal was captured at 2000 Hz from the left index finger. (**D,E**) PPG processing: PPG signals were processed using Kubios 3.1.0 to detect beat times. (**F**) Beats were assigned to pre- and post-stimulus intervals, then inter-beat intervals (IBIs) were computed between successive beats and assigned to the time point of the second beat. (**G,H**) Pre- and post-stimulus IBIs were combined to construct a heart rate change time course with respect to the mean pre-stimulus heart rate. (**J**) Group-mean HR change time courses were computed for each stimulus and the overall post-stimulus peak deceleration, tps_max_, is determined. HR change at tps_max_ of each time course was then combined with machine learning predictions as fixed effects of a general linear mixed-effects model (GLMM) where random slope and intercept effects were modeled subject-wise.

The purpose of this task was to induce affect processing concurrently with fMRI and psychophysiological signal acquisition for subsequent construction, independent validation, and comparison of predictive measures of affect processing. Specific details of this task were previously reported in Bush *et al*.^30^ with the following summary of the task design provided for experimental context. Ninety image stimuli were selected from the International Affective Picture Set (IAPS) to maximally span the normative affect representation subspace. Image stimuli were presented for 2 s (stimulation) followed by a visual fixation cross presented for a random inter-trial interval (ITI) sampled uniformly from the range of 2–6 s. IAPS image presentations were balanced across two 9.25 min scan runs according to the images’ normative valence and arousal scores in order to minimize the correlation of general linear model regressors that would be used to identify their functional neural activations by post-hoc analysis. The image order was then fixed for all participants. A graphical representation of the image stimuli and their presentation scheme is depicted in Fig. 1, panel A.

### MR image acquisition and preprocessing

We acquired all imaging data using a Philips 3 T Achieva X-series MRI scanner (Philips Healthcare, Eindhoven, The Netherlands) with a 32-channel head coil. MR acquisition parameters for both anatomic and functional images used for both INCA and CTM studies have been reported previously in Bush *et al*.^30^. Standard image correction and preprocessing pipelines, previously detailed in Bush *et al*.^30^, were applied to both anatomic and functional images. In brief, we acquired anatomic images with a MPRAGE sequence (matrix = 256 × 256, 220 sagittal slices, TR/TE/FA = 8.0844/3.7010/8°, final resolution = 0.94 × 0.94 × 1 mm^3^). We acquired functional images with EPI sequence parameters: TR/TE/FA = 2000 ms/30 ms/90°, FOV = 240 × 240 mm, matrix = 80 × 80, 37 oblique slices, ascending sequential slice acquisition, slice thickness = 2.5 mm with 0.5 mm gap, final resolution 3.0 × 3.0 × 3.0 mm^3^. We processed all MRI data using AFNI (Version AFNI_18.2.06)^39^ unless otherwise noted. Anatomical data were processed via skull stripping, spatial normalization to the icbm452 brain atlas, and segmentation via FSL^40^ into white matter (WM), gray matter (GM), and cerebrospinal fluid (CSF). Functional images underwent the following processing sequence: despiking; slice-time correction; deobliquing; motion correction; transformation to the spatially normalized anatomic image; regression of the mean timecourse and temporal derivative of the WM and CSF masks as well as a 24-parameter motion model^41,42^; spatial smoothing (6-mm FWHM Gaussian kernel); and scaling to percent signal change. Gray matter (GM) masks for each subject were created using anatomical segmentation. A group-level GM mask was constructed incorporating only GM voxels present in ≥50% of the individual subject GM masks. The group-level GM mask was used to report group-level neural activation localizations related to the fit hyperplanes (see *2.12 Construction of Neuroanatomical Encoding Parameters)*.

### BOLD beta-series construction

As reported in Bush *et al*.^30^ we exploited the beta-series method^43^ to construct whole-brain, task-related neural activation maps corresponding to each individual IAPS image. To summarize, we constructed a general linear model (GLM) that includes hemodynamic regression functions for each individual experimental stimulus as well as additional functions related to motion and drift artifacts. We then solved this GLM, and the resulting parameter set associated with each stimulus regressor constitutes the neural activations induced by that stimulus. These neural activations served as features on which to conduct MVPA as depicted in Fig. 1, panel B.

### Heart rate acquisition and preprocessing

We recorded psychophysiological measures of heart rate using a BIOPAC MP150 Data Acquisition System (BIOPAC Systems Inc., Goleta, CA) combined with the AcqKnowledge software and TSD200-MRI pulse photoplethysmogram (PPG), which has been shown to detect inter-beat intervals with accuracy comparable to electrocardiogram^44^. The PPG was placed on the left index finger. Respiration and skin conductance response were also recorded for all subjects. For those subjects that were part of the CTM study (n = 31), facial electromyography was concurrently recorded. This work explores the PPG data only.

Using Kubios heart rate variability analysis software (version 3.1.0), we processed all of the raw PPG signals using the automatic correction parameters for artifact removal and exported the processed data as a Matlab structure (Fig. 1, Panel D). We then extracted the heart beat times detected by Kubios from this structure for the remaining analysis (Fig. 1, panel E).

### Heart rate change analysis

As there is not a consensus methodology by which to analyze heart rate change induced by affective image stimuli, we extracted from the literature a set of thematic methodological elements that mapped onto our experiment design, allowing us to first reproduce and then extend prior work. Prior research has often focused on the comparison between distributions of HR changes induced by subsets of IAPS images representing the polar-extremes of positive and negative valence. Prior methods of analysis have also centered on the time course of HR change post-stimulus onset. HR change, typically, is measured in units of deceleration from rest where the resting heart rate is estimated over a time period occurring immediately prior to stimulus onset or from a separate resting state portion of the experiment^7,17,18^. HR change is also often reported in units of average beats per unit time (e.g., beats per minute, bpm), which is the inverse of the inter-beat interval (IBI), the time elapsing between successive heart beats. Our methodology has attempted to honor all of these methodological conventions while remaining grounded within the principles of data-driven prediction-based analysis.

#### HR change time course construction

We computed time courses of HR changes with respect to the System Identification Task’s inter-trial intervals (brief periods of rest uniformly randomly sampled from the range 2–6 s). Pre-stimulus beats were identified within a one second interval pre-stimulus onset^9,10^. Post-stimulus beats were identified within a four second interval post-stimulus onset (depicted in Fig. 1 panel F). Pre- and post-stimulus beats were converted to IBIs by calculating the difference between the times of successive beats, using, if necessary, beats falling outside the strict time intervals to form a beat pair. We assigned the IBI value to the time of the second beat of the pair. We then computed the average over the pre-stimulus IBIs to use as a baseline for determining HR deceleration post-stimulus onset.

We formed time courses of HR change by calculating, at each half-second post-stimulus, the current heart rate relative to the pre-stimulus average heart rate^9^. To construct these half-second bpm estimates, we identified the time-fractionally-weighted average of each IBI that fell within the half-second interval of interest, converted this average from IBI (s) to bpm and then subtracted the mean pre-stimulus average heart rate (converted to bpm). By performing this computation for half-second intervals spanning 0–4 s post-stimulus onset, we formed smooth time courses of HR change for each IAPS stimulus for each subject (depicted graphically in Fig. 1, panel H).

#### Identifying the time of measurement for HR deceleration

One challenge for our methodology was determining a point at which to measure deceleration. The literature provides a wide range of parameters. We settled on the following methodology (driven by our data). First, we compute the group mean time-course for each of the 90 IAPS stimuli. We then divide these data into two sets, positive and negative stimuli (using the normative valence scores of the stimuli as the label and partitioning based on the middle Likert score of 5). Based on widely reported patterns of deceleration of negative stimuli^7,13,16–18^ we used the time-point of the minimum of the mean trajectory over the negative stimuli as the point at which to measure stimuli (i.e., maximum deceleration, denoted tps_max_; see Supplemental Fig. S1).

### Intra-subject multivariate regression training and cross-validation

We conducted multivariate pattern analysis (MVPA) using a linear support vector machine (SVM) regression architecture^45^, fitrsvm, parameterized by the default settings of the Matlab Statistics Toolbox^46^. We chose the SVM architecture for this analysis based on its established performance in domains having small sample sizes^47^, fast analytical solution, and previously demonstrated ability to predict normative valence scores from high-dimensional GM neural activations^30^. The architecture was trained according to intra-subject (i.e. within subject) leave-one-out-cross-validation (LOOCV). That is, for a set of subjects, N, and feature-label pairs, M, the j^th^ prediction (j ∈ M) for the i^th^ subject (i ∈ N) was made using an SVM regression model trained on the M-1 disjoint feature-label pairs (k ∈ M-1; k ≠ j). All beta-series features were GM masked to the individual subject, i.

### Mixed-effects modeling

We estimated prediction effect sizes of our affective measures via general linear mixed effect model (GLMM). The measure of interest was set to be the normative valence scores of the experimental stimuli. Affective measures (i.e., HR deceleration and SVM-predicted valence) were incorporated as fixed effects. Random slope and intercept effects were modeled subject-wise. GLMMs were solved using Matlab’s fitlme function. Effect-sizes (R^2^) were calculated from the resultant model residuals. The significance of the random effects was tested by constructing two models for each test (including and excluding the random effects) and comparing the fit of the model variants using a likelihood ratio test (implemented as Matlab’s compare function). Only significant random effects were incorporated into estimates of effect size.

### Controlling for stimulus set affective valence via neutral stimuli thresholding

One challenge for the analytical methodology employed in this work, which arises from our IAPS image sampling process (see *Section 2.3 System Identification Task* as well as Fig. 1, panel A), was that our selected image set exhibited normative affect rating scores that maximally (and continuously) spanned the arousal-valence plane. Therefore, our stimulus set provided us with no clear demarcation between polar-extreme positively and negatively valent categories by which to group the concurrent HR changes. Further, a review of the literature found no accepted methodology on which to base the determination of positive and negative valence. Indeed, prior studies used clusters of polar-extreme positively or negatively valent stimuli rather than a natural demarcation, such as the middle Likert score of the IAPS image set (i.e., valence = 5). A summary of the valence and arousal scores exhibited by image sets employed in prior HR change analyses are provided in Supplemental Table S1.

To address this confound in the literature, we performed our primary analysis across a range of stimulus sets in which we incrementally remove stimuli from a region of neutrality (symmetrically about the middle Likert score) in order to control the polar-extremity of the positively and negatively valent stimuli in the modeled stimulus set. Starting with the baseline condition (all stimuli), we classified the image stimuli according to their pre-defined normative valence scores (calculated as mean Likert scores from a 9 point scale) relative to the middle Likert score (5). We then conducted GLMM modeling of SVM and ΔHR fixed effects for the stimulus set as well as secondary calculations of the mean and standard deviation of the normative scores of the positively and negatively valent stimuli as well as the fraction of stimuli kept (relative to the baseline condition).

We then repeated this modeling process at small increments of the threshold used to classify positively and negatively valent stimuli as follows. For each *threshold* value (on the range, 0–3, at increments of 0.2) we stratified the stimuli to exclude those falling within a normative valence score range of 5+/−*threshold*. We then labeled those stimuli falling outside the threshold as positively or negatively valent.

## Results

### Reproducing canonical HR change in response to affectively valent stimuli

We qualitatively reproduced the structure of valence-specific HR deceleration in response to IAPS stimuli within the fMRI environment. Consistent with prior studies^13–18,38^, we report a relatively large post-stimulus HR deceleration for negatively valent stimuli, depicted graphically in Supplemental Fig. S1. However, in contrast with earlier work^7,15^ we report neither deceleration nor a triphasic response to positively valent stimuli.

### Reproducing canonical functional neuroanatomical encoding of affective valence

We found significant inter-analysis consistency between the affective valence encodings detected in this work and the encodings detected in earlier work that used a substantially smaller (n = 19) set of subjects^30^. Encoding parameters derived from the intra-subject MVPA models fit in this experiment explain 85% of the variance of those prior encoding parameters and no encoding parameter (out of 2524 voxels that survive joint-analysis permutation testing) changed sign, depicted graphically in Supplemental Fig. S2.

### Neural activation patterns are stronger measures of affective valence induction than heart rate change

Both HR change (p = 0.036; F-test; null: β = 0) and SVM-predicted valence (p < 0.001; F-test; null: β = 0) significantly contribute fixed effects to the GLMM prediction of normative valence scores of the stimuli, as depicted in Supplemental Fig. S3. Random effects were not significant. Moreover, SVM-predicted valence contributed an order of magnitude more explanation of variance (R^2^ = 0.049) in comparison to the contribution of HR change (R^2^ = 0.001).

### Neural Activation patterns measure valence induction across a broad range of stimulus sets

**Figure 2:**
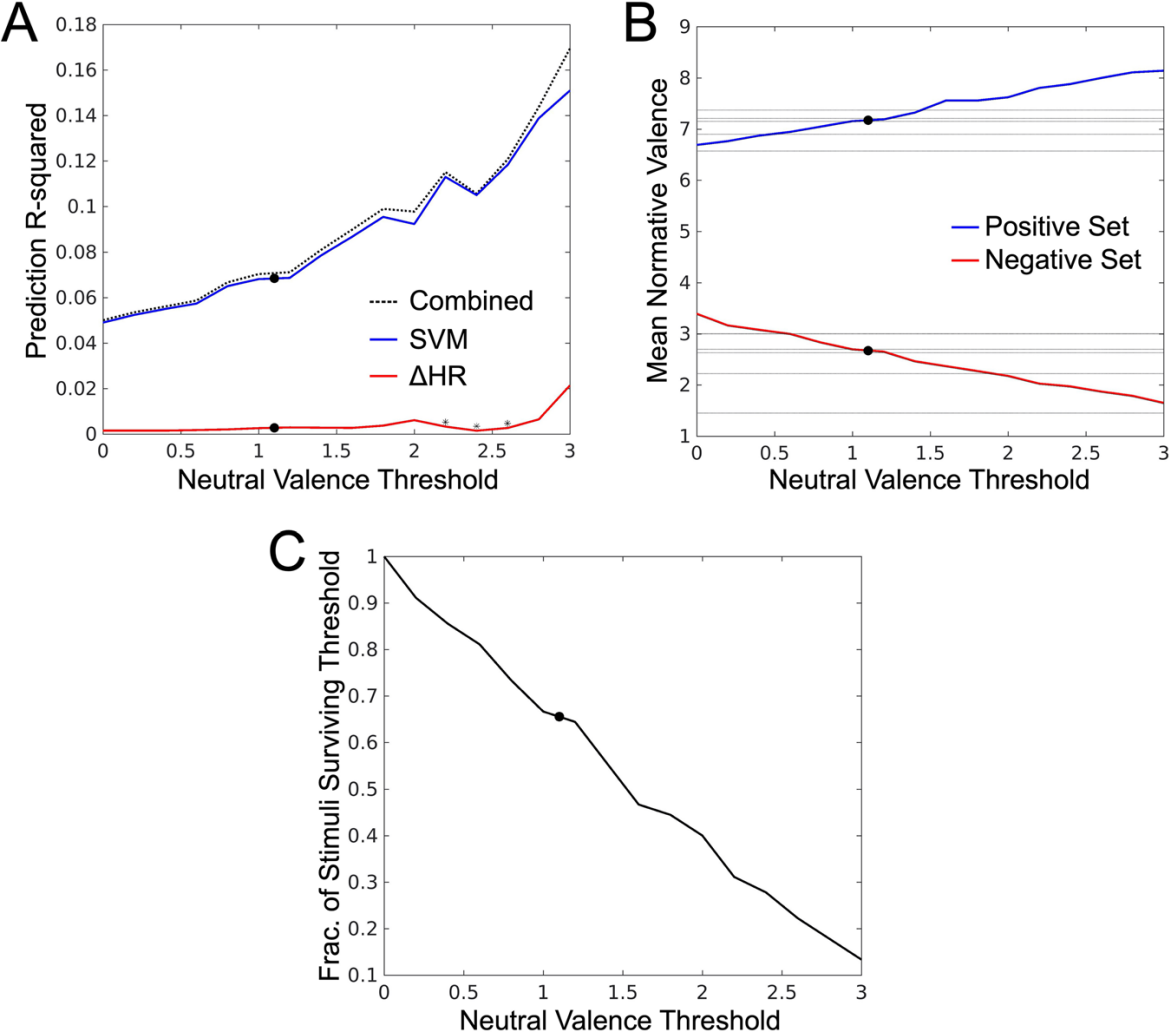
Summary of the effects of polar-extremity in the normative valence scores of the stimulus on modeling performance. (**A**) Estimation of the contribution of fixed effects, separately for heart rate deceleration and SVM predictions as a function of the width of the threshold used to exclude “neutral” stimuli. This threshold is reported in units of the 9-point Likert scale within which the IAPS image set was originally acquired. *Denotes a non-significant fixed effect at that level of thresholding. (**B**) Mean normative valence scores of the positively and negatively valent stimulus sets created by excluding neutrally valent images as a function of the exclusion threshold. Note, the black dots depict the mean normative valence scores of the positively and negatively valent image sets analyzed in Bradley *et al*.^7^. The neutral valence threshold representative of this stimulus set in our dataset was transferred to panels A and C, respectively, for interpretation of these findings. Also note, horizontal gray lines denote mean valence scores for positively and negatively valence stimulus sets reported for other prior work reporting heart rate change discrimination of valenced image stimuli (see Supplemental Table 1 for reference). (**C**) Fraction of full stimulus set included as a function of scaling.

A critical limitation in relating these combined prediction findings to the broader literature linking HR change to affective valence is the selection of stimuli, which varies widely in the literature. To control for this potential confound, we repeated the GLMM modeling reported in Section 3.3 along a continuously varying set of stimuli (see Materials and Methods Section 2.12) in which the most neutrally valent stimuli (according to IAPS normative valence ratings) are excluded from analysis, thus generating stimulus sets of incrementally greater polar-extremity. The resultant prediction effect sizes and related analyses are depicted in Fig. 2. In support of the findings in Section 3.3, SVM-predicted valence contributes more to the explanation of variance than does heart rate change at every level of thresholding (Fig. 2, panel A). With a complex feature space such as the brain, the stronger SVM-prediction compared to heart rate change is not surprising. Moreover, this difference between SVM prediction is robust to methodological differences of stimulus selection that may exist between this work and related reports in the literature.

As would be expected, based on how the base stimulus set maximally spans the arousal-valence plane of normative affect scores (see Fig. 1, panel A), mean valence of the resulting sets of positively and negatively valent stimuli scales linearly with thresholding (see Fig. 2, panel B); moreover, the scaling rate is dictated by sign (positive stimulus sets become more positively valent with thresholding and vice versa). In combination with the increases in prediction efficacy shown in Fig. 2 (panel A), this scaling suggests that neural activation patterns also become more consistently predictive at polar-extremes of affective valence, which confirms (in a regression modeling paradigm under highly controlled thresholding) an earlier report of this phenomenon in a classification paradigm^30^. It is also useful to note that in Fig. 2 (panel C), the threshold that achieves comparable polar-extremes of valence to the experiment reported in Bradley *et al*.^7^ requires removal of approximately 33% of our study’s stimuli, supporting the conceptualization of the middle third of the IAPS image set as neutrally valent, which comports with an intuitive, but unreported, tertiary categorization of IAPS stimuli in the affective valence dimension.

### Heart rate change and neural activations uniquely predict affective valence

A critical inference to be drawn from Fig. 2 (panel A) is that HR change remains a weak, but reliable predictor of affective valence across a broad range of stimulus sets. To understand the unique contribution of HR change we attempted to predict, via GLMM, SVM-based predictions using ΔHR-based prediction (see Supplemental Fig. S4). This test did not yield a significant model (p = 0.07; F-test; null: β = 0). Further, the normative valence score of the stimulus is highly predictive of both the SVM-based prediction error (p < 0.001; F-test; null: β = 0) and ΔHR-based prediction error (p < 0.001; F-test; null: β = 0); moreover, ΔHR-based prediction error is a strong predictor of SVM-based prediction error (p < 0.001; F-test; null: β = 0), depicted in Supplemental Fig. S5. Combined, these findings suggest that HR change, with respect to MVPA, captures unique information concerning the induction of affective valence in our experiment.

## Discussion

The primary aim of this study was to extend the comparison of the relative validity of physiological and neural measurements to the domain of affective valence induction. A key contribution of this study is that the comparison was performed on a stimulus set representing a broad continuum of conveyed affective valence, i.e., including a range of neutral and weakly valent stimuli in addition to the polar-extremes of positively and negatively valent stimuli as have been commonly reported in the heart rate change literature. Another contribution of this work is that we demonstrated a significant empirical relationship between the neural activations derived in this study with an earlier effect-size comparison between physiological and neural measurements of affective arousal induction. We believe this type of analysis and reporting will contribute to better generalization of our findings to multiple, independent dimensions of affect processing. Finally, to our knowledge, this is the first study to explore the combined predictive effect of physiological and neural measurements and the first to clearly rank their individual contributions in predicting normative valence scores of IAPS stimuli.

### Limitations

The combined predictions of HR change and neural activations explained less than 6% of total variance of the normative valence scores of our stimulus set (up to approximately 17% of total variance for a highly thresholded, and therefore, valently polar-extreme stimulus set). We attribute part of the remaining unexplained variance to the demographics of our sample. Previous research showed that IAPS normative scores may differ depending upon a participant’s age^48–50^ as well as their sex^51–53^. As the age and sex of our sample differs from the sample from which the IAPS normative valence scores were initially derived^54^, we anticipate that individual variation in affect processing strategies and their neural activations (with respect to the IAPS image stimuli included in this study) contributes to unexplained variance, a study limitation that could have been addressed by collecting each subject’s self-reported affect stimulus rating scores.

We also suspect that our experiment design, which was not optimized for post-hoc HR change analysis, may also contribute to unexplained variance. Our inter-trial intervals of 2–6 s were not sufficient for all subjects to fully re-acquire basal HR and neural activations. Therefore, when measuring HR deceleration relative to a baseline HR estimated from a short pre-stimulus interval, the baseline HR may be influenced by residual responses related to the affective response to the previous stimuli. Our use of beta-series to construct SVM predictions of valence, however, attenuates the contribution of prior stimuli to stimulus-bound neural activations, likely contributing to some of the differences in prediction effect-size between the measures by introducing a bias towards the neural activation measure. Within this same line of reasoning, it is possible that our stimulus presentation time was not sufficient to optimize HR changes. Prior work has shown that the length of stimulus presentation (on the range of 0.03 to 12 s) alters HR deceleration^17^. This could potentially weaken the predictive effect-size of HR changes with respect to neural activation; however, we point out that the beta-series method captures the affective content of the stimuli, making it suitable for these relatively fast presentation formats. Further, temporally structured artifacts in the BOLD signal, notably the effect of respiration not specifically attributable to motion^55^, may also contribute to prediction effect size differences in our measurement modalities and were not specifically modeled in our MRI processing pipeline.

Finally, we acknowledge that the fit MVPA models may be exploiting aspects of the neural activations induced by the image stimuli that are incidentally correlated with the valence property of affect based on biases in the IAPS dataset (e.g., colors and shapes typical of infant photos being overly represented within images labeled as inducing positive affect). We previously explored this possibility in Bush *et al*.^30^, and found no direct evidence for these correlations. Rather, Bush *et al*.^30^ showed that SVM model encodings of affective properties were highly reproducible across affect induction experiments which differed by both image stimuli and subjects. However, the definitive test of MPVA-based prediction of affect processing prediction remains to be reported and represents an ongoing goal for this research team.

### Future directions

**Figure 3:**
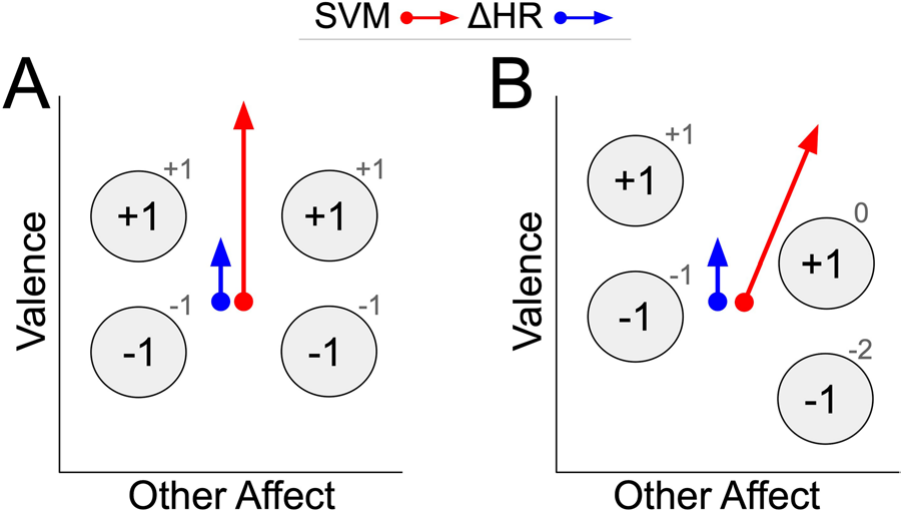
Conceptual model of relative contributions of neuroimaging and physiological measures of affect induction based on a theory of individual differences. Patterns of neural activation are represented as gray circles. Normative valence scores of the stimuli associated with these neural activations are presented as labels within the circles’ boundaries. Single subject perceived valence scores of the stimuli are depicted as gray numbers above and to the right of the circles. **(A)** Theoretical Case 1: a subject’s perceived valence of the stimuli exactly agrees with the normative valence scores. The resulting direction of prediction for fixed effects of heart rate deceleration (blue arrow) and predictions of a fit support vector machine (red arrow) mutually agree. **(B)** Theoretical Case 2: a subject’s perceived valence of the stimuli (as indicated by shifts of neural activations and associated perceived valence scores) disagrees with the normative valence scores, thereby increasing the relative predictive effect of heart rate deceleration. When image stimuli are polar-extreme, perceived affect better aligns with the stimulus set’s normative affect scores (panel A); therefore, measures of both neural activation (which is fit to, and, therefore, predicts normative affect scores) and HR change (which, in theory, measures perceived affective valence) predict along the axes of normative valence scores (the measure of interest in this experiment). However, when image stimuli are neutral, disagreements between the subjects’ perceived affect and the normative valence scores emerge. Measures of neural activation generalize to these individual differences during the learning process whereas HR change continues to weakly, but reliably, predict perceived affect, yielding relatively lower predictive effect size on the measure of interest.

Prior work^30^ presented evidence that neural activations for affectively polar-extreme stimuli exhibit greater inter-subject consistency than less polar-extreme stimuli. Following this line of evidence, we formed a theoretical model describing the relationship between the prediction effect-size of HR change and individual differences in affect processing, depicted graphically in Fig. 3. This model potentially explains how measurements of HR change and neural activations uniquely and significantly inform the combined prediction of the stimuli’s normative valence scores. In sum, measurement modalities differ in their detection of disagreements between a subject’s perceived affect and the normative valence scores, which are most likely to emerge among neutrally valent stimuli^30^. Measures of neural activation generalize to these individual differences during the learning process whereas HR change continues to weakly, but reliably, predict perceived affect, yielding relatively lower predictive effect size on the measure of interest. An experiment design which captures an additional measure of affective induction (e.g., self-reported scores of each stimulus or an additional physiological measure of valence, such as facial EMG) could test this theoretical model. Data collection is ongoing to power such an experiment.

## Conclusions

This study characterized, for the first time, the combined effects conveyed by both patterns of neural activation and heart rate deceleration in predicting normative valence scores of affectively valent image stimuli. Both measures were shown to exhibit significant and unique predictive effects; however, patterns of neural activation were shown to explain a significantly greater proportion of variance than heart rate deceleration. These observed traits (significance and uniqueness as well as relative performance) persisted as the positive and negative polar-extremity of the stimuli’s valence were incrementally increased to represent the diversity of stimulus sets reported in the heart rate deceleration literature. In sum, these findings support the acquisition of heart rate deceleration concurrently with fMRI to provide convergent validity of induced affect processing in the dimension of affective valence.

## Supplementary information

Supplementary information.
