# Supplementary material for: Combining Physiological and Neuroimaging Measures to Predict Affect Processing Induced by Affectively Valent Image Stimuli
Source: Sci Rep. 2020 Jun 9;10:9298. doi: 10.1038/s41598-020-66109-3 (PMC7283349; doi:10.1038/s41598-020-66109-3)
Supplement: Supplementary file 1 — Supplementary information. [file 41598_2020_66109_MOESM1_ESM.docx]

**SUPPLEMENTAL MATERIALS**

Combining Physiological and Neuroimaging Measures to Predict Affect Processing Induced by Affectively Valent Image Stimuli.

Kayla A. Wilson, G. Andrew James, Clint D. Kilts, Keith A. Bush^*^

Brain Imaging Research Center, University of Arkansas for Medical Sciences

***Correspondence:**Keith A. Bush

kabush@uams.edu

**Keywords**: heart rate change, affect, emotion, valence, MVPA, fMRI, IAPS

**Materials and Methods**

*Construction and Comparison of Functional Neuroanatomical Encoding Parameters.*

Our exploitation of linear SVM permitted us to readily generate, via the Haufe-transform (Haufe et al., 2014; Hebart, et al., 2015), neural encoding parameters from our fit decoding model. We previously reported our methodology for performing this voxelwise transformation in Bush et al. (2018b). These encoding parameters allowed us to identify those neuroanatomical regions that activated in concordance with the modeled quantities, e.g. normative valence score. Further, we assessed the statistical significance of the encoding parameters through the application of permutation testing (Schreiber & Krekelberg, 2013). Unlike univariate methods (where each voxel is modeled as a separate hypothesis test), our linear SVM modeling approach applies a single, whole-brain (GM) hypothesis test to decode the relevant neuroactivation patterns. Due to the mathematical structure of the linear SVM, decoding parameters may covary, which must be corrected for during statistical significance testing. Permutation testing allowed us to estimate the null hypothesis of our model class via sampling.

For clarification, the computed whole-brain encoding parameter set is tested for significance against the null distribution of whole-brain encoding parameter sets (n=1200 random permutations). We formed this null distribution by uniformly randomly distributing the labels of the feature-label pairs used to train the SVMs. We then determined the fraction of encoding parameters in the null distribution that are observed to be more extreme than the observed parameter (group mean encoding parameter) along each dimension of the model (i.e., voxel location), keeping only those parameters for which the fraction, f, is sufficiently small (f<0.025, 2-sided test at p<0.05).

We then calculated those voxels which jointly survive permutation testing of both the INCA study previously reported in Bush et al. (2018) and the current study which combined subjects from INCA as well as CTM. We then conducted general linear modeling to measure the agreement in encoding where the measure of interest were the encoding values computed in Bush et al. (2018) and those computed in the current study.

*Encoding Parameters*

The authors have made the encoding parameters and gray matter mask necessary to reproduce this analysis publicly available on the Open Science Framework: <https://osf.io/6fdzu/>

**Results**


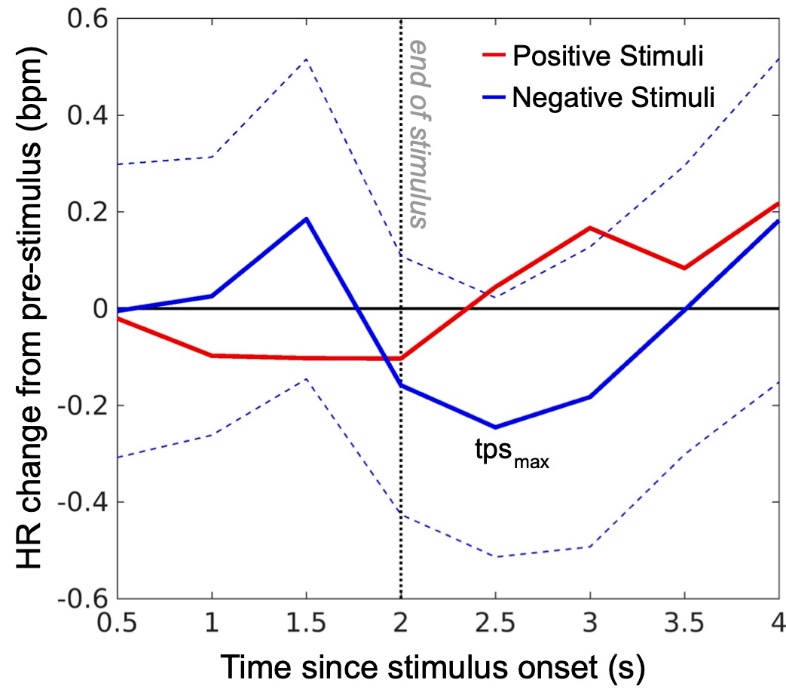


Figure Supplemental S1:

Group mean stimulus-induced heart rate change timecourses relative to pre-stimulus (resting) heart-rate for the affective image stimuli. Bold red line depicts group mean HR change time course in response to positively valent stimuli. Bold blue line depicts group mean HR change time course in response to negatively valent stimuli. Dashed blue lines depict the 95% confidence intervals of the negatively valent stimuli mean time courses. The dotted vertical line denotes the end of the image stimulus presentation (2 s post-stimulus onset). Maximum heart rate deceleration in response to negatively valent stimuli is labeled, tps_max._


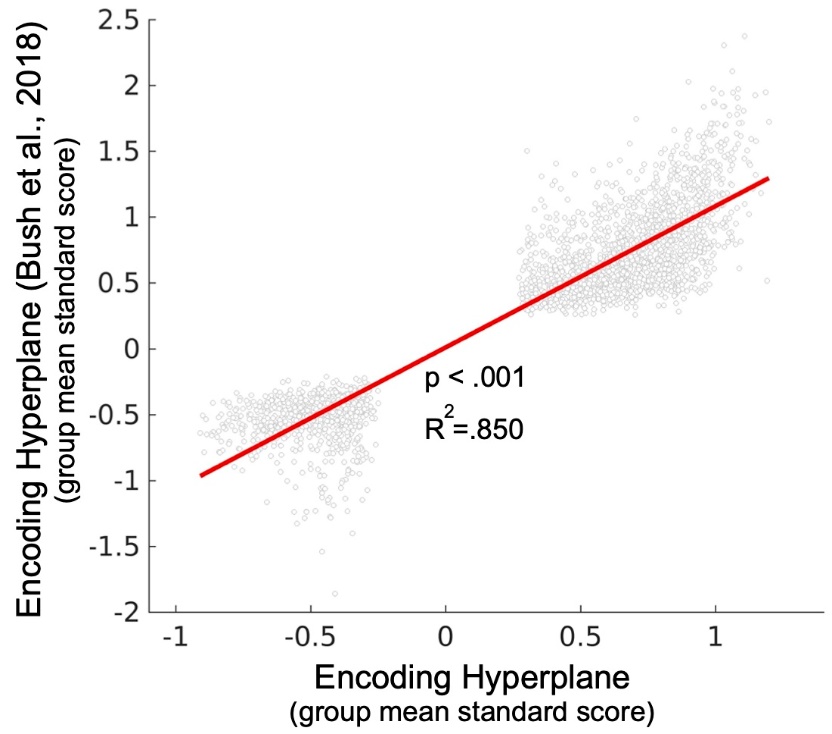


Figure Supplemental S2:

Summary inter-analysis consistency of fMRI-derived group-level gray matter mean intra-subject encodings (Haufe et al., 2014) of affective valence. Gray circles depict encoding values calculated for this study (combined CTM and INCA study data) and the INCA study alone (Bush et al., 2018) for voxels that jointly survived permutation testing (p<0.05). Reproduction (85% variance explained) of the encoding hyperplane provides evidence for the robustness of the reported MVPA modeling approach and suggests that the findings reported here augment prior findings which identified consistency between neuroimaging and physiological measures of affective arousal (Bush et al., 2018).


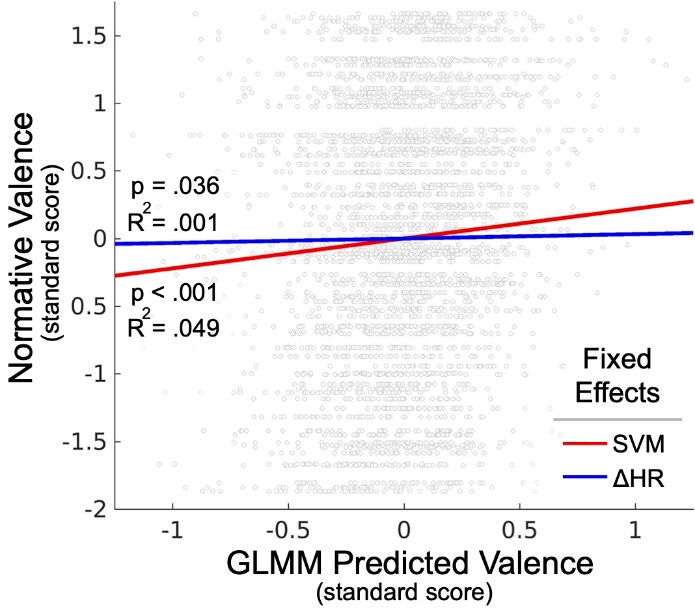


Figure Supplemental S3:

Summary of general linear mixed-effects model (GLMM) to compare affect induction measurements. Grey circles represent GLMM model predictions of valence (for combined fixed effects, excluding non-significance random effects) of all stimuli compared to the normative valence scores of those stimuli (horizontal repeated measures denote individual subjects). Bold lines depict the individual fixed effect contributions of support vector machine predictions (SVM, plotted in red) and heart rate change (ΔHR, plotted in blue). P-values denote the significance of each fixed effect within the GLMM. Reported R^2^ values represent the variance explained by the individual fixed effect.


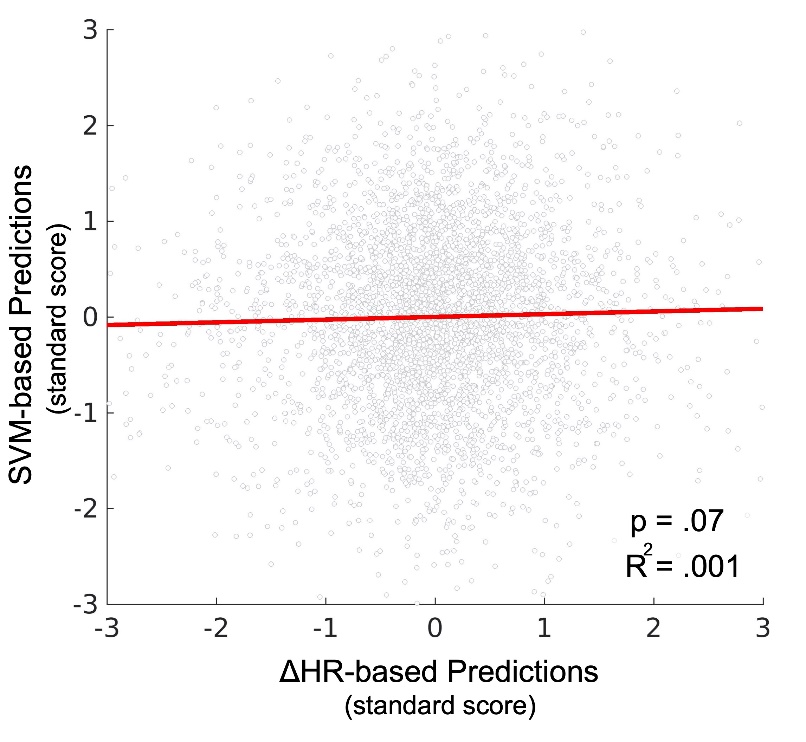


Figure Supplemental S4:

GLMM test of the relationship between physiological and neuroimaging based measures of affect induction. The measure of interest was SVM prediction of the normative valence scores of the affective stimuli. The fixed effect was heart rate deceleration (ΔHR). Slope and intercept effects were modeled subject-wise. The fixed effect (β=.03) was found to be not significant (p=0.07; F-test; null: β=0). Random effects were not significant.


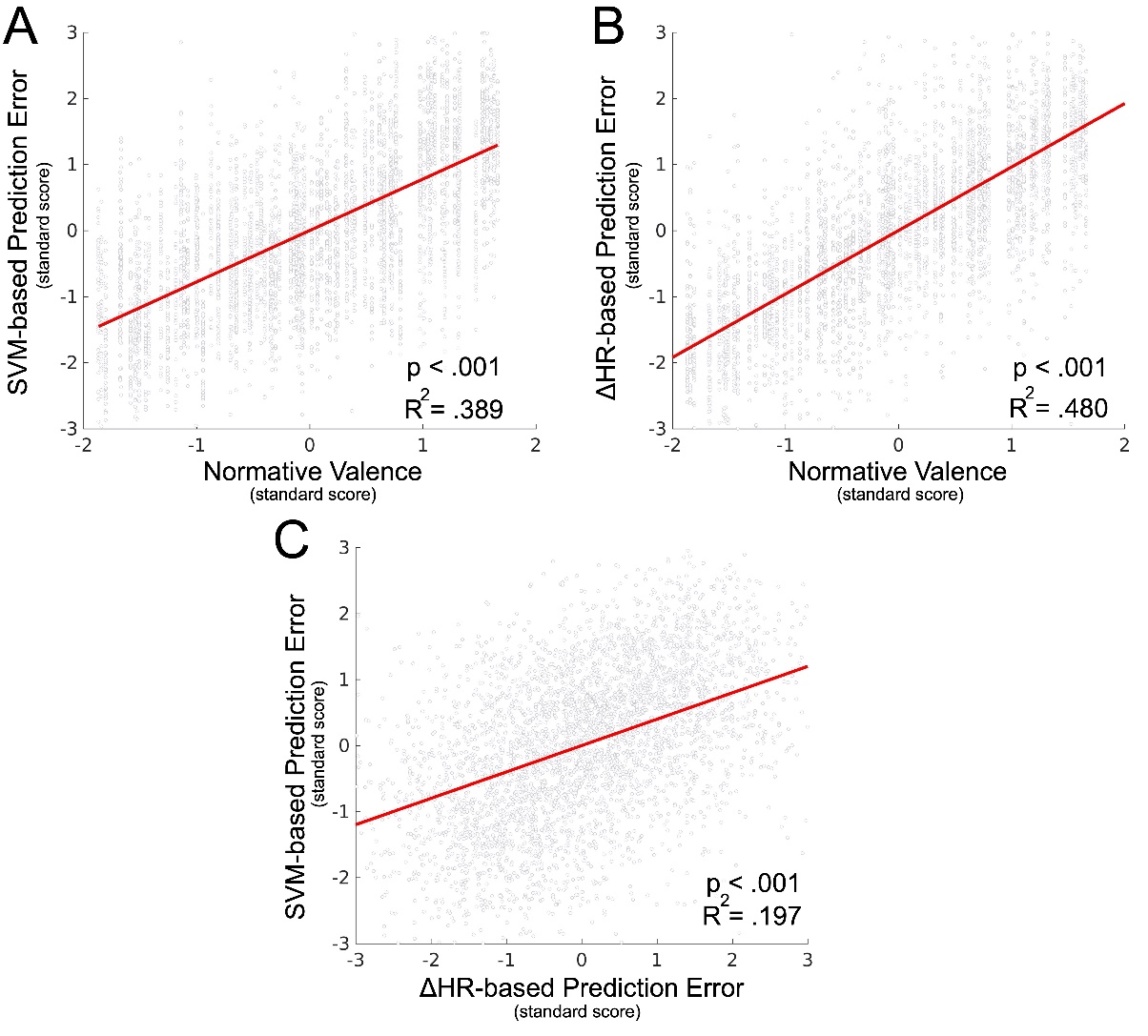


Figure Supplemental S5:

GLMM tests of the relationship between normative valence scores of the stimuli and physiological and neuroimaging based measures of affect induction. For all models, slope and intercept effects were modeled subject-wise. **(A)** The measure of interest was SVM prediction residuals. The fixed effect was the normative valence scores of the affective stimuli (converted to standard score). The fixed effect (β=.78) was found to be significant (p<0.001; F-test; null: β=0). Random effects were not significant. **(B)** The measure of interest was heart rate deceleration (ΔHR) prediction residuals. The fixed effect was the normative valence scores of the affective stimuli (converted to standard score). The fixed effect (β=.96) was found to be significant (p<0.001; F-test; null: β=0). Random effects were not significant. **(C)** The measure of interest was SVM prediction residuals. The fixed effect was heart rate deceleration prediction residuals. The fixed effect (β=.40) was found to be significant (p<0.001; F-test; null: β=0). Random effects were not significant.

| Affect Property Summary of Positively and Negatively Valent Image Subsets | | | | | | |
| --- | --- | --- | --- | --- | --- | --- |
|  | Subset | Arousal | | Valence | | |
| Study | Neg/Pos | Mean (SD) | Mean (SD) | |  |  |
| This study | Negative | 5.06 (1.45) | 3.39 (1.11) | |  |  |
|  | Positive | 4.84 (1.35) | 6.69 (0.94) | |  |  |
| Bradley et al., 2001^♦^ | Negative** | 4.70 (1.43) | 2.63 (0.80) | |  |  |
|  | Positive | 5.30 (1.48) | 7.21 (1.43) | |  |  |
| Katahira et al., 2014 | Negative | 5.26 (0.79) | 2.70 (0.36) | |  |  |
|  | Positive | 5.06 (0.71) | 7.37 (0.59) | |  |  |
| Kuniecki, et al., 2003^♦^ | Negative | 6.95(0.38) | 1.45(0.09) | |  |  |
|  | Positive*** | 6.05(0.61) | 6.57(0.55) | |  |  |
| Pollatos et al., 2006^♦^ | Negative | 5.69 (1.35) | 3.00 (0.90) | |  |  |
|  | Positive* | 5.30(0.98) | 7.15 (0.85) | |  |  |
| Ruiz-Padial et al., 2011 | Negative | 6.92 (0.68) | 2.22 (0.91) | |  |  |
|  | Positive | 6.71 (0.40) | 6.90 (0.55) | |  |  |
| Urry et al., 2009 | Negative | 6.05 (0.77) | 2.12 (0.47) | |  |  |

Table Supplemental S1:

*A single positive image listed as 2203 in Pollatos et al. (2006) was not present in the IAPS imageset. However, its absence could not significantly change the mean and standard deviation of the positive subset.

**Image 3330 listed in Bradley et al. (2001) was not present in the IAPS imageset. A personal communication with Bradley corrected the image to 3300.

***These values are reported for the combined male and female normative scores. However, Kuniecki et al. used only male subjects and the positive valent images are drawn exclusively from erotica.

^♦^In these studies, when referencing the IAPS database for the valence and arousal values there were two sets of scores for images 4220 and 1590, representing repeated presentations in the original acquisition of scores. As the image number does not indicate the presentation order, the set of scores were averaged together for our calculations.
